# ‘Seaweed appearance’ in squamous cell carcinoma of the penis: A new dermoscopic finding

**DOI:** 10.1002/ski2.275

**Published:** 2023-08-15

**Authors:** Wei Zhang, Lingxi Gu, Jianglong Feng, Hui Fan, Hongguang Lu

**Affiliations:** ^1^ Department of Dermatology Affiliated Hospital of Guizhou Medical University Guiyang Guizhou China; ^2^ Department of Pathology Affiliated Hospital of Guizhou Medical University Guiyang Guizhou China

## Abstract

Squamous cell carcinoma of the penis is an uncommon cancer. Vascular feature on dermoscopy is common in all forms of invasive squamous cell carcinoma, and the presence of the specific vascular features is often used to aid diagnosis. Here, we reported a new dermoscopic finding—seaweed‐like vascular pattern in squamous cell carcinoma of the penis.

## Graphical abstract

A new dermoscopic finding—seaweed appearance in squamous cell carcinoma of the penis.

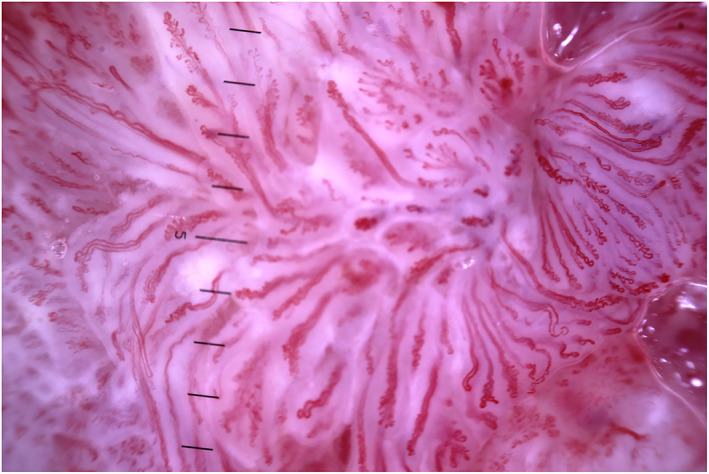

A 48‐year‐old man presented with 2‐year history of asymptomatic red plaque on his penis (Figure 1a) (a). Dermoscopy revealed polymorphous vessels (including linear, serpentine, helical, coiled morphology) arranged radially in a pattern that resembles floating red seaweed (Figure 1b) (b). Histologic examination showed atypical squamous cells invading into the dermis, with some keratin pearls and multiple dysmorphic intratumoral vessels (Figure 1c) (c). The diagnosis of invasive cutaneous squamous cell carcinoma (SCC) was made.^1^ The unusual vascular pattern in dermoscopy like red seaweed is rare in penile SCC, probably because of dilated and convoluted capillaries in dermis of the genital mucosa and tumour‐associated angiogenesis^2^

**FIGURE 1:**
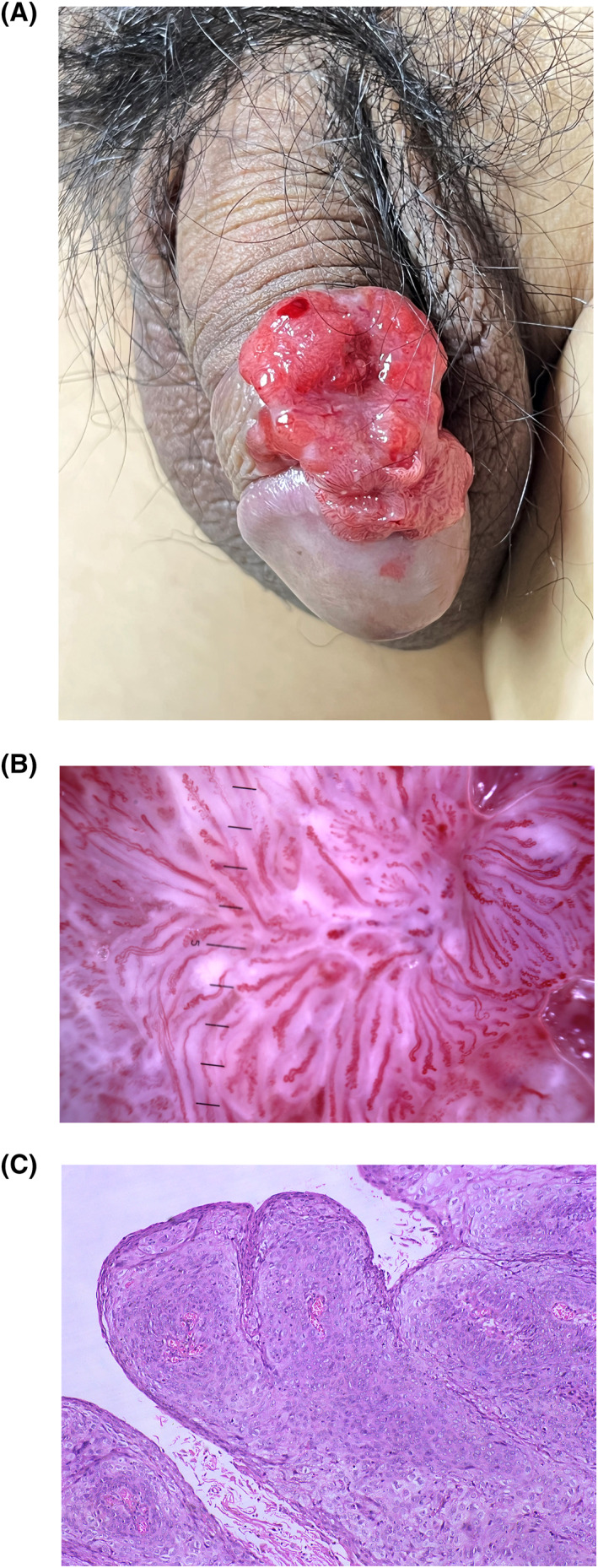
Clinical manifestation, dermoscopic feature, and histological analysis. (a) Clinical manifestation. Red plaque was observed on his penis. (b) Dermoscopic feature. Dermoscopy of the lesion surface showed polymorphous vessels arranged radially in a pattern that resembles floating red seaweed. (c) Histological examination. Atypical squamous cells invading into the dermis, with some keratin pearls and multiple dysmorphic intratumoral vessels (Haematoxylin‐eosin stain; original magnification 10X.).

## CONFLICT OF INTEREST STATEMENT

None declared.

## AUTHOR CONTRIBUTIONS

**Wei Zhang**: Conceptualization (Lead); Data curation (Lead); Resources (Lead); Visualization (Lead); Writing – original draft (Lead). **Lingxi Gu**: Data curation (Equal); Investigation (Equal); Resources (Equal); Visualization (Equal). **Jianglong Feng**: Data curation (Equal); Resources (Equal); Visualization (Equal). **Hui Fan**: Supervision (Equal); Writing – review & editing (Equal). **Hongguang Lu**: Conceptualization (Supporting); Supervision (Equal); Writing – review & editing (Supporting).

## ETHICS STATEMENT

Not applicable.

## Data Availability

Data sharing is not applicable to this article as no new data were created or analyzed in this study.
